# Involvement of Three Esterase Genes from *Panonychus citri* (McGregor) in Fenpropathrin Resistance

**DOI:** 10.3390/ijms17081361

**Published:** 2016-08-19

**Authors:** Xiao-Min Shen, Chong-Yu Liao, Xue-Ping Lu, Zhe Wang, Jin-Jun Wang, Wei Dou

**Affiliations:** Key Laboratory of Entomology and Pest Control Engineering, College of Plant Protection, Southwest University, Chongqing 400716, China; sxmshenxiaomin@163.com (X.-M.S.); leochongyu@163.com (C.-Y.L.); luxueping91@163.com (X.-P.L.); zhewangswu@163.com (Z.W.); wangjinjun@swu.edu.cn (J.-J.W.)

**Keywords:** *Panonychus citri*, carboxylesterase, fenpropathrin resistance, heterologous expression, RNA interference

## Abstract

The citrus red mite, *Panonychus citri* (McGregor), is a major citrus pest with a worldwide distribution and an extensive record of pesticide resistance. However, the underlying molecular mechanism associated with fenpropathrin resistance in this species have not yet been reported. In this study, synergist triphenyl phosphate (TPP) dramatically increased the toxicity of fenpropathrin, suggesting involvement of carboxylesterases (CarEs) in the metabolic detoxification of this insecticide. The subsequent spatiotemporal expression pattern analysis of *PcE1*, *PcE7* and *PcE9* showed that three CarEs genes were all over-expressed after insecticide exposure and higher transcripts levels were observed in different field resistant strains of *P. citri*. Heterologous expression combined with 3-(4,5-dimethyl-thiazol-2-yl)-2,5-diphenyltetra-zolium bromide (MTT) cytotoxicity assay in *Spodoptera frugiperda* (Sf9) cells revealed that *PcE1*-, *PcE7*- or *PcE9*-expressing cells showed significantly higher cytoprotective capability than parental Sf9 cells against fenpropathrin, demonstrating that PcEs probably detoxify fenpropathrin. Moreover, gene silencing through the method of leaf-mediated dsRNA feeding followed by insecticide bioassay increased the mortalities of fenpropathrin-treated mites by 31% (*PcE1*), 27% (*PcE7*) and 22% (*PcE9*), respectively, after individual *PcE* gene dsRNA treatment. In conclusion, this study provides evidence that *PcE1*, *PcE7* and *PcE9* are functional genes mediated in fenpropathrin resistance in *P. citri* and enrich molecular understanding of CarEs during the resistance development of the mite.

## 1. Introduction

The citrus red mite, *Panonychus citri* (McGregor) (Acari: Tetranychidae), is one of the most important citrus pests responsible for significant economic losses [1,2]. It feeds on more than 112 different plant species [3]. Heavy infestations lead to leaf drop, twig dieback, and fruit drop, and all negatively affect citrus yield and quality. A short life-cycle and high reproductive rate has allowed *P. citri* to rapidly develop resistances to many insecticides and acaricides [4,5,6,7]. To date, the citrus red mite ranks the third among species that evolved severe resistance from the family *Tetranychidae* [3].

Pyrethroids insecticides, analogues naturally occurring pyrethrins extracted from dried flowers *Chrysanthemum cinerariaefolium*, have been widely used in the control of mites and pests, contributing to more than 25% of world insecticide sales for their high efficiency, broad-spectrum and relatively low toxicity [8,9,10,11]. However, extensive and widespread use of pyrethroids has led to pest resistance as a consequence, which impedes pest control efforts [12]. Knock down resistance (kdr) mutation on target genes and elevated activity of detoxification enzymes are two crucial mechanisms to confer high resistance to pyrethroids [13,14,15,16]. The point mutation F1538I in segment 6 of domain III from sodium channel gene, which is known to confer strong resistance to pyrethroids, has been confirmed from comparison between resistant and susceptible strains of *Tetranychus urticae* [17]. A recent study about the citrus red mite revealed that a Phe1538 to Ile mutation from the sodium channel played a crucial role in fenpropathrin resistance after comparison of field fenpropathrin-resistant (WZ) and susceptible strains [18].

Carboxylesterases (CarEs) belong to a superfamily of multifunctional enzymes ubiquitous in most living organisms, including animals, plants and microbes. Insect CarEs are mainly involved in insecticide resistance or hormone and semiochemical metabolism [19,20]. As one of the most crucial metabolic detoxification systems in insects, CarEs have been shown to be associated with development of resistance to many insecticides including pyrethroids through gene amplification, improvement of mRNA stability and point mutation [19,21]. Overexpressed esterases are invovled in fenvalerate resistance of *Helicoverpa armigera* [22] and lambda-cyhalothrin resistance of *Aphis glycines* [23]. Similar studies have also been found in mites and ticks. For instance, there was a correlation between esterase activity and bifenthrin resistance in *T. urticae* [24] and significant elevation of esterase activity existed in λ-cyhalothrin-resistant populations of *Rhipicephalus bursa* [25].

Understanding the expression profiles of a detoxifying gene and characteristics of its recombinant protein is crucial to clarify the function of the gene related to insecticides-detoxifying process, but limited data is available in *P. citri*. Our previous study found the elevated expression of *PcGSTm5* in abamectin resistant strain and its sensitive response to abamectin exposure, indicating that *PcGSTm5* might be involved in abamectin resistance [26]. Meanwhile, the synergist TPP dramatically increased the toxicity of fenpropathrin, indicating that CarEs-mediated detoxification was probably an important mechanism of *P. citri* to pyrethroids resistance. In this study, to better understand the underlying molecular mechanism of CarEs-mediated pyrethroids resistance in *P. citri*, a series of experiments employing biochemical and molecular approaches were conducted. We first sequenced, phylogenetically analyzed and characterized the spatiotemporal expression pattern of two novel CarE genes, *PcE7* and *PcE9*, along with another previously isolated gene *PcE1* (GenBank number: GQ144324). The function study combined with cytotoxicity assays using Sf9 cells overexpressing *PcE1*, *PcE7* and *PcE9* were subsequently investigated. In addition, the reverse genetic study through the method of leaf-mediated dsRNA feeding was applied to explore the link between the CarE genes and pyrethroids resistance in *P. citri*.

## 2. Results

### 2.1. Synergism Studies

To investigate the effect of esterases on resistance of *P. citri* against the insecticide fenpropathrin, the synergist triphenyl phosphate (TPP) was used here and the mite of Beibei (BB) population was chosen for bioassay (Table 1). After the application of TPP, the LC_50_ decreased from 4.339 mg/L to 1.405 mg/L and the synergism fold amounted to 3.09, suggesting that CarEs played a crucial role in detoxification process of *P. citri* to fenpropathrin.

### 2.2. Characterization and Phylogenetic Analysis

**Figure 1:**
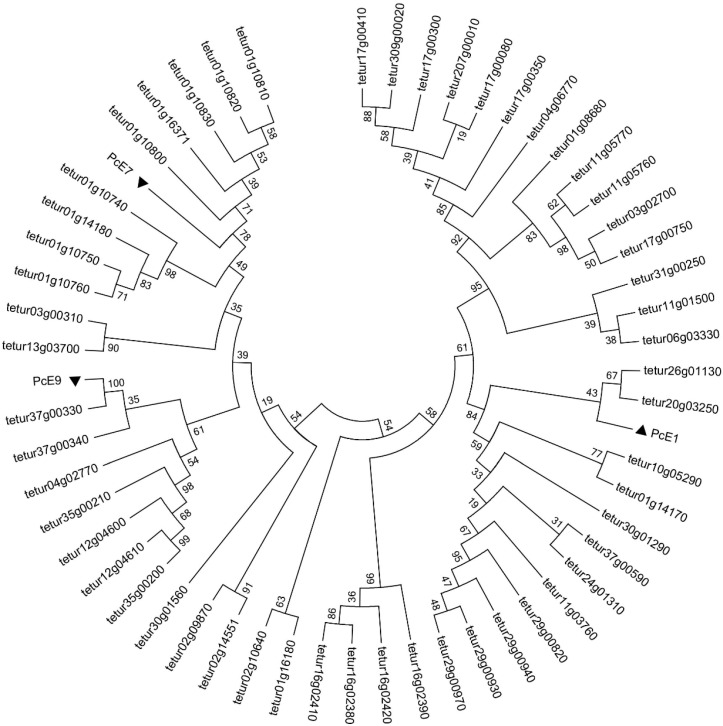
Rooted phylogenetic tree of three CarEs from *Panonychus citri* with CCE proteins from *Tetranychus urticae.* All the protein sequences of the *T. urticae* CCEs were download from http://bioinformatics.psb.ugent.be/orcae/overview/Tetur and *PcE1, PcE7* and *PcE9* were retrieved from the National Center for Biotechnology Information. All the amino acid sequences were aligned using ClustalW, and a distance neighbor-joining tree was generated using MEGA 5.0. The three CarEs genes examined in this study are marked with triangles.

Besides *PcE1*, isolated from our previously reported transcriptome [27], two novel CarE genes (*PcE7* and *PcE9*) were cloned, respectively. No point mutation occurred among different field strains of *P. citri*. The cDNAs encoded proteins of 569 (*PcE7*, GenBank accession number: JQ951938), and 566 (*PcE9*, GenBank accession number: JQ951939) amino acid residues. Both *PcE7* and *PcE9* contained all the conserved motifs for maintenance of CarEs including the catalytic triad and oxyanion hole. The phylogenetic trees revealed the evolutionary relationships between the three esterases of *P. citri* as well as their relationships with 55 CarEs from *T. urticae* (Figure 1). For *PcE1*, there was a close clustering within clade J′ of the neurodevelopmental class from *T. urticae* and PcE1 shared an overall amino acid identity of 54% with tetur20g03250. Both *PcE7* and *PcE9* were assigned to clade J″ of the neurodevelopmental class, with identities of 52% between PcE7 and tetur01g10820 and 95% between PcE9 and tetur37g00330, respectively.

### 2.3. Expression Profile to Fenpropathrin Exposure

**Figure 2:**
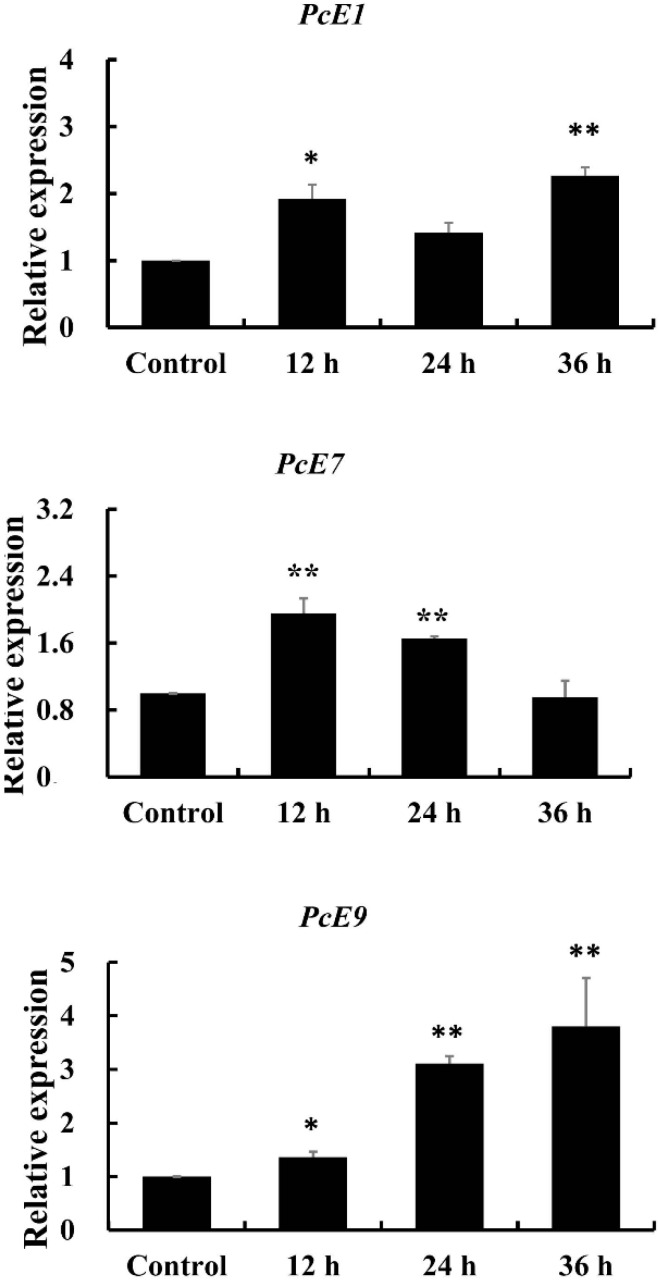
Expression patterns of *PcE1*, *PcE7* and *PcE9* in response to fenpropathrin exposure. Relative expression levels were calculated based on the control, which was defined as a basal value of 1. The vertical bars indicated standard errors of the mean (*n* = 3). The asterisk on the vertical bars indicate significant differences in the mRNA level of between control and treatment (Student’s *t*-test, * *p* < 0.05 and ** *p* < 0.01).

The mRNA expression levels of *PcE1*, *PcE7* and *PcE9* were all significantly increased after mite exposure to a sub-lethal concentration of fenpropathrin (LC_30_, 0.818 mg/L) in a time-dependent manner (Figure 2). A consistent increase in *PcE9*, expression level occurred after fenpropathrin treatment. In contrast, *PcE7* responded to fenpropathrin stress more quickly while the up-regulation alleviated at 36 h treatment. Among the three genes, the expression pattern of *PcE1* fluctuated little, ranging from 1.92-fold at 12 h exposure to 2.26-fold at 36 h exposure.

### 2.4. Different Strains Expression Profiles

**Figure 3:**
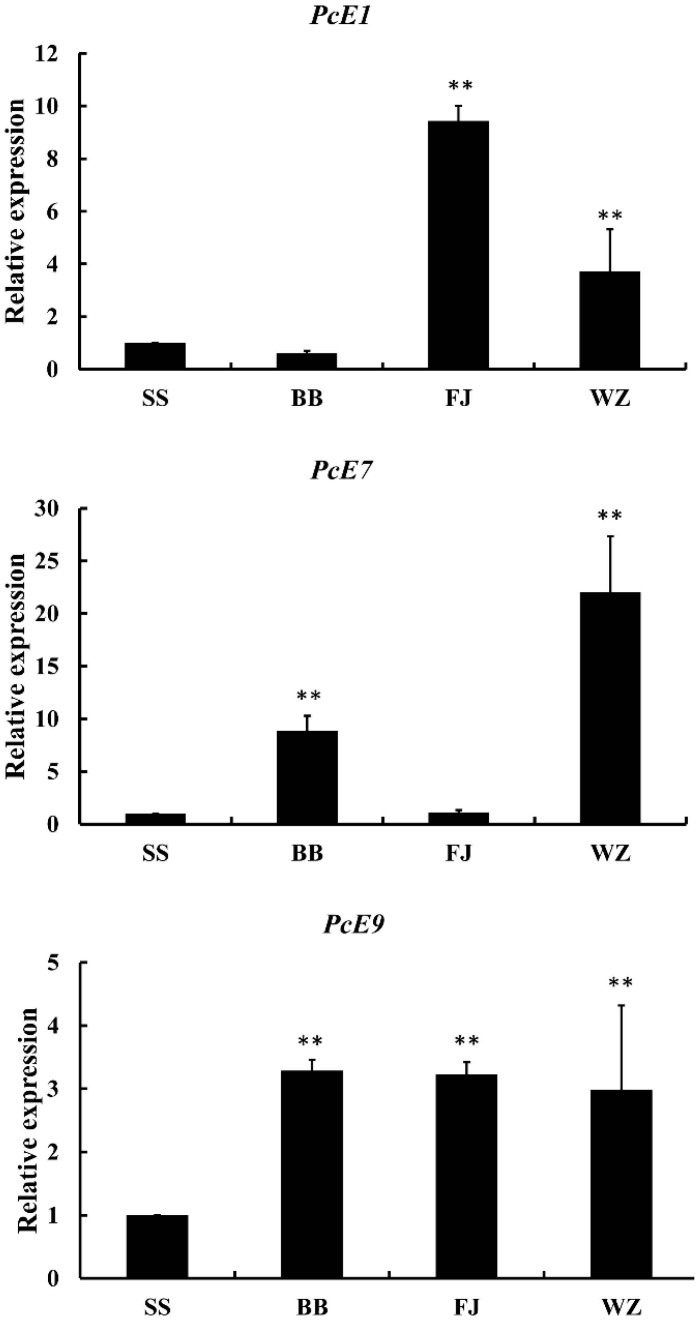
Expression patterns of *PcE1*, *PcE7* and *PcE9* in different populations. Relative expression levels were calculated based on the control (SS), which was defined as a basal value of 1. The vertical bars indicated standard errors of the mean (*n* = 3). The asterisk on the vertical bars indicate significant differences in the mRNA level of between control and treatment (Student’s *t*-test, ** *p* < 0.01).

The expression patterns of *PcE1*, *PcE7* and *PcE9* from *P. citri* of different field strains were shown in Figure 3. Compared to the counterparts of SS, generally higher expression levels of three genes were recorded from different field strains. For *PcE1*, WZ and FJ strains indicated 9.43- and 3.72-fold increase, respectively. *PcE7* expressed a dramatic fluctuation among the different field strains, ranging from 23.03-fold in WZ to 1.12-fold in FJ. The expression ratio of *PcE9* fluctuated least among three strains, while significantly higher compared to that of SS.

### 2.5. Enzyme Activity of Recombinant Enzymes with Sf9 Cells

**Figure 4:**
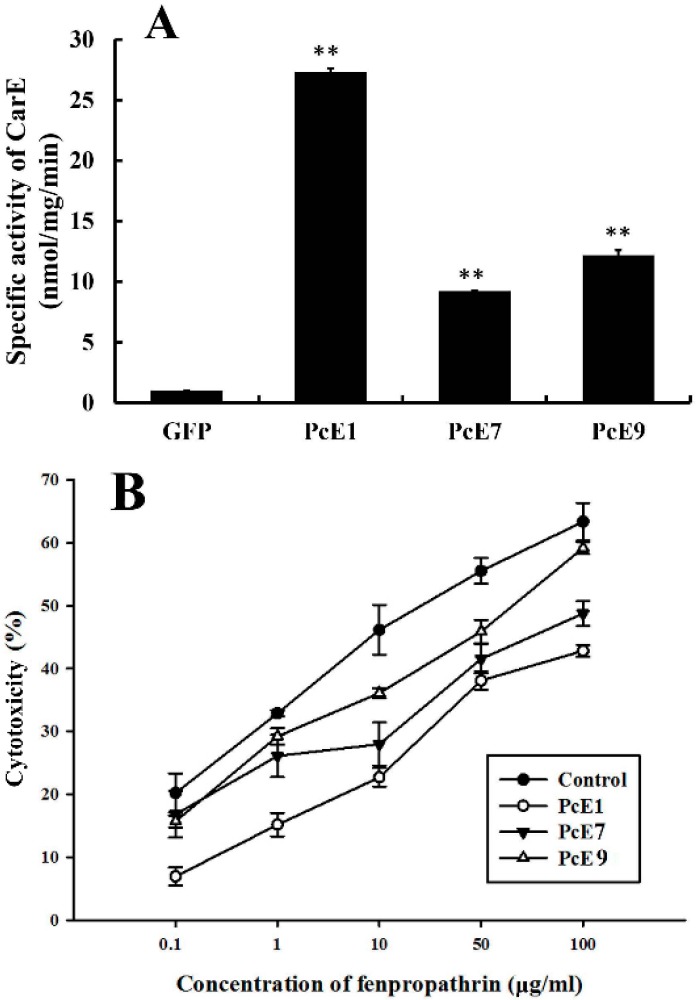
Specific activity of carboxylesterase (CarE) in recombinant enzymes expressing *PcE1*, *PcE7* or *PcE9* toward the substrate of α-NA (**A**); and cytotoxicity of *PcE1-*, *PcE7-*, *PcE9-* and EGFP-expressing cells against fenpropathrin (**B**). The percentage of viable cells was detected using 3-(4,5-dimethyl-2-yl)-2,5-diphenyltetrazolium bromide cytotoxicity assays. Data are means ± SE of three independent experiments. Asterisks (*) above the error bars indicate statistical differences determined by the independent samples *t*-test (** *p* < 0.01).

An enzymatic assay of the recombinant *PcE* protein was measured in vitro to determine if there was CarE-specific activity (Figure 4A). The results showed that compared with the GFP-expression protein, the CarE-specific enzyme activities of *PcE1*, *PcE7* and *PcE9* toward the substrate of α-naphthyl acetate (α-NA) were 27.31-, 9.21- and 12.17-fold higher, respectively (*p* < 0.01).

### 2.6. Cytotoxicity Assay

The cytotoxicity assay with 3-(4,5-dimethyl-thiazol-2-yl)-2,5-diphenyltetra-zolium bromide (MTT) were performed to examine the toxicity of fenpropathrin in Sf9 insect cells expressing *PcE1*, *PcE7* or *PcE9* (Figure 4B). The value of the median lethal concentration (LC_50_) was calculated from a plot of percentage of cell viability against different concentrations of fenpropathrin by Probit assay. The results revealed that there were higher cell viability against cytotoxic effects of fenpropathrin in *PcE1*-, *PcE7*- or *PcE9*-expressing cells than that in the enhanced green fluorescent protein (*GFP*)-expressing cells. LC_50_ values were recorded in *PcE1-* (258.30 μg/mL), *PcE7-* (265.10 μg/mL) and *PcE9*- (258.30 μg/mL) expressing cells against fenpropathrin, all about 10-fold of that in *GFP*-expressing cells (26.04 μg/mL).

### 2.7. Susceptibility of Mites to Fenpropathrin after RNAi of CarE

**Figure 5:**
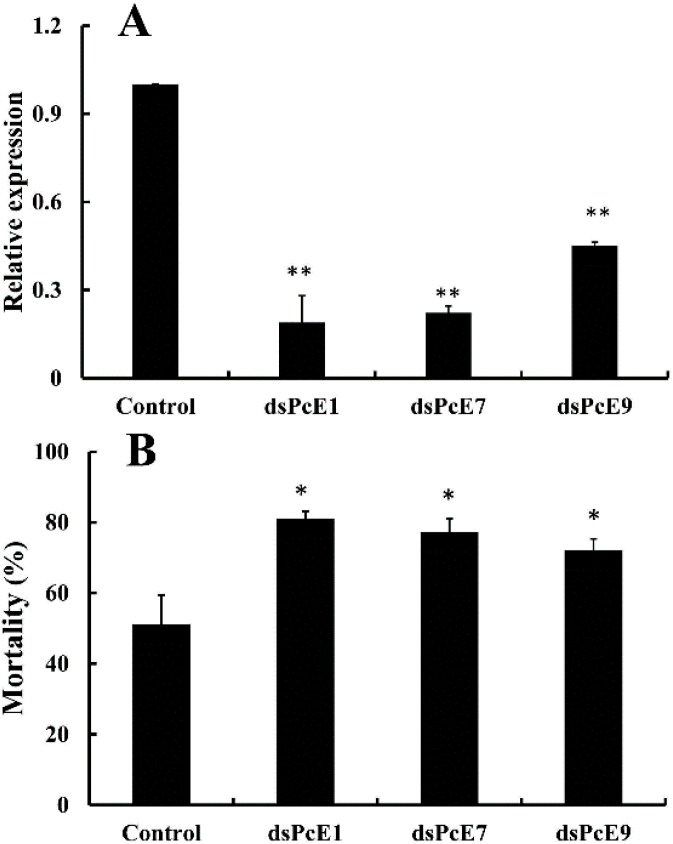
Susceptibility of *Panonychus citri* to fenpropathrin after silencing of *PcE1, PcE7* and *PcE9* by RNA interference. (**A**) Silencing efficiency of *PcE1, PcE7* and *PcE9* after *P. citri* were investigated 48 h after the gene silencing treatment; (**B**) The mortalities of the citrus red mites were investigated after the fenpropathrin treatment at the concentration of LC_50_ (4.519 mg/L). Results were mean ± SE of four biological replication (*n* = 4). The asterisk on the vertical bars indicate significant differences in the mRNA level of the three genes or the mortalities between control and treatment (Student’s *t*-test, * *p* < 0.05 and ** *p* < 0.01).

After RNAi by the plant leaf method, RT-qPCR was applied to investigate the knock-down efficiency of the *CarE* genes expression in *P. citri* of the field strain BB. The results showed that the transcript levels of *PcE1*, *PcE7* and *PcE9* were significantly decreased 81%, 77% and 54%, respectively, compared with control dsGFP (Figure 5A). The results demonstrated that the transcripts of the *CarE* genes were successfully silenced with RNAi in *P. citri*. Subsequently, the sensitivity of mites after RNAi to fenpropathrin were detected. When treated with LC_50_ of fenpropathrin, the mortality of mites after *dsPcE1*, *dsPcE7* and *dsPcE9* treatments increased significantly by 31%, 27% and 22%, respectively (Figure 5B).

## 3. Discussion

CarEs play important physiological roles in detoxification of xenobiotics and resistance to insecticides in insects. They have been reported in metabolic resistance to pyrethroids in several insect species and mites [22,28,29,30]. The synergist (TPP) is normally considered as the inhibitor of esterases. Through synergist experiments, we can obtain preliminary evidence of the relationship between insecticide resistance and detoxification pathways. The current bioassay found the synergist TPP dramatically increased the toxicity of fenpropathrin, indicating that CarEs-mediated detoxification was probably an important mechanism of pyrethroid resistance in *P. citri*. In the current study, besides *PcE1* that was identified to participate in the detoxification of acaricides [27], two novel CarE genes (*PcE7* and *PcE9*) were chosen from transcriptome data as the candidate genes based on the qPCR results of their over-expression in fenpropathrin-resistant filed strains of *P. citri*. The subsequent phylogenetic analysis with CarE genes from *T*. *urticae* indicated that *PcE1* was clustered into clade J′ while *PcE7* and *PcE9* were assigned to clade J″ of the neurodevelopmental class.

Point mutations within genes that determine substrate specificities, as well as elevation of CarEs activity arising from transcription or gene amplification, are predominantly two molecular basis of CarEs-mediated resistance in target insects [31,32]. The oxyanion hole mutation (G137D) resulted in modest levels of resistance of *Lucilia cuprina* to a range of diethyl organophosphorus insecticides (OPs) [33]. Two site mutations (K14Q and N354D) of *CarEs* with high frequency were found to be involved in cotton aphid resistance to malathion [32]. In this study, sequence alignment of three genes (*PcE1*, *PcE7* and *PcE9*) found no point mutation occurred among different field strains of *P. citri*. It is no surprise considering the modest resistance (ranging from 7.7- to 21.7-fold) of the mite strains in the paper.

Over-expression of CarEs results from up-regulated transcription of a single copy or accumulation of multiple copies of the esterase genes [34]. The up-regulation of two α-esterase genes mediated metabolic resistance to malathion in the oriental fruit fly, *Bactrocera dorsalis* [35]. The increased transcription levels and gene copy numbers of CarE were responsible for malathion resistance of the cotton aphid [32]. In mites, there are similar records, for example, esterases were proved to be important metabolic factors involved in resistance of *T. urticae* against abamectin [36]. Metabolic resistance against pyrethroids mediated by CarEs was also well documented [37] and increased esterase activities were observed in bifenthrin-resistant strains of *T. urticae* [38]. In *T. cinnabarinus*, an enhanced activity of esterases correlated to mite resistance against abamectin and fenpropathrin and *TCE2* was over-produced in fenpropathrin-resistant strain of the mite [39]. In addition, *TCE2* was inducible when exposure to the acaricide, indicating the potential involvement of *TCE2* in *T. cinnabarinus* resistance to fenpropathrin. Transcript profiling analysis revealed that three *CarEs* genes were all significantly elevated in a time-dependent manner after mite exposure to fenpropathrin. Compared to *PcE9*, *PcE1* and *PcE7* responded more quickly to fenpropathrin stress in a short-term treatment. A consistent increase in *PcE9* transcript level was observed, suggesting the gene probably played a crucial role in a long-term acaricide exposure. The up-regulation of esterase genes provide options for the development of resistance, representing a general xenobiotic detoxification response [40].

For enzyme characteristics studies, heterologous expression is an efficient approach to obtain target gene products and provides chances to explore gene functions in vitro. Therefore, the recombinant CarEs including PcE1, PcE7 and PcE9 were expressed in Sf9 cells and their enzymatic properties were characterized. All recombinant proteins showed significant catalytic activities when α-NA was used as the substrate. A distinct activity toward the conjugates of glutathione and 1-chloro-2,4 dinitrobenzene were recorded for the recombinant protein of PcGSTm5 expressed in *Escherichia coli*, and the kinetic characters of expression product were systematically investigated [26]. In *T. cinnabarinus*, *TCE2* gene was successfully expressed by *E. coli* expression system, and subsequent biochemical analysis found the recombinant protein presented 2-fold of the activity of the crude enzyme extracts [41]. As insecticides have previously been reported to express cytotoxic effects, such as oxidative stress in Sf9 cells [35], treatment with fenpropathrin in Sf9 cells can cause cell mortality unless cells are protected by detoxification or sequestration of fenpropathrin. Thus, cell-based inhibition assays employing MTT cytotoxicity assays were conducted to further clarify the detoxification capabilities of recombinant CarEs to fenpropathrin. The treatment of fenpropathrin in Sf9 cells caused cell mortality to different extent. The higher LC_50_ values were observed in PcE1-, PcE7- and PcE9-expressing cells than that in the control GFP-expressing cells to fenpropathrin exposure, indicating that the recombinant CarEs can protect cells from cytotoxicity of fenpropathrin. Similar results have also been reported in many other insect species. For instance, flavonoids greatly elevated sensitivity of CYP6AA3- and CYP6P7-expressing Sf9 cells to cypermethrin toxicity, due to inhibition effects on mosquito enzymes [42]. In *Anopheles minimus*, CYP6P7- or CYP6AA3-expressing cells showed higher detoxification capabilities than parental Sf9 cells against cytotoxicity of pyrethroids [43]. Heterologous expression combined with MTT assay revealed the detoxification role of BdCarE4 and BdCarE6 against malathion [35].

RNAi technique was further applied to evaluate possible roles of *PcEs* in fenpropathrin-resistance of *P. citri*. The LC_50_ for BB field strain were used to detect the effect of RNAi on the change of sensitivity of *P. citri* to fenpropathrin. The results indicated that transcript levels of *PcE1*, *PcE7* and *PcE9* were all successfully knocked down by feeding dsRNA of individual *PcE* gene to the mites from BB strain. The similar method via leaf-mediated dsRNA delivery has been used in *T. urticae*, *T. cinnabarinus* and whiteflies [41,44]. In *P. citri*, though the time-dependent profile of RNAi efficiency was not investigated, a high RNAi efficiency (at least >54%) was recorded at 24 h after dsRNA feeding in this study. These results indicated that the RNAi system applied in this study was useful for gene function research of the citrus red mite. The subsequent bioassay data showed that those mites after feeding dsRNA-PcE exhibited significantly higher susceptibility when exposed to fenpropathrin, suggesting that gene silencing decreased the detoxification capabilities of CarEs encoded by *PcEs* on fenpropathrin. As an effective method to determine gene function, RNAi possesses the potential for application in pest management in the field because of its high specificity and has been employed in many insects and mites [45,46,47]. The total CarE activity in *Aphis gossypii* decreased significantly after dsRNA-CarE treatment and the susceptibility to omethoate was suppressed in individuals of the resistant aphid strains [48]. The transcript levels of *TCE2* in resistant strains of *T. cinnabarinus* were effectively silenced after RNAi and the following bioassay results suggested that the resistant levels of the mite to several acaricides were significantly decreased after the down-regulation of *TCE2* [41]. The current bioassay data showed that higher mortalities were recorded after leaf-mediated dsRNA feeding of individual gene, further supporting the link between the expression of *PcEs* and fenpropathrin resistance.

## 4. Experimental Section

### 4.1. Mites

A laboratory colony of *P. citri*, which was originally collected from the citrus nursery without pesticide application for more than 10 years at the Citrus Research Institute of the Chinese Academy of Agricultural Sciences, served as the relatively susceptible strain (SS). This strain was found to be susceptible to fenpropathrin based on results of laboratory bioassays and was reared without the exposure to any acaricides. It had been maintained at 25 ± 1 °C and 60% relative humidity under a 14:10 h light:dark condition. Three fenpropathrin-resistant strains were collected in 2015 from the citrus orchards in Beibei (BB), Wanzhou (WZ) and Fengjie (FJ) districts, Chongqing, China, respectively. Previous bioassay results showed that three field strains of *P. citri* have developed about 7.7- (BB), 57.7- (WZ) and 21.7-fold (FJ) resistance to fenpropathrin compared to that of SS.

### 4.2. Bioassays and Fenpropathrin Exposure

Bioassays was conducted using the leaf-dip method as described previously [41]. Leaf disks with a diameter of 25 mm were made from fully expanded lemon leaves and washed with nuclease-free water (Promega, Fitchburg, MA, USA) before use, and placed on a water-saturated sponge in Petri dishes (9 cm in diameter). The wet sponge was covered with a piece of thin absorbent paper to prevent mites from escaping. Thirty female adults were transferred onto a leaf disk with a soft brush. Then the leaf disks with mites were dipped 5 s into serial dilutions of fenpropathrin with acetone served as control. Triton-100 (0.1% *v*/*v*) (Beijing Dingguo Chang Sheng Biotech Co., Ltd., Beijing, China) was used as surfactant in all the solution. Subsequently, the leaf disks with mites were incubated under climate-controlled conditions at 25 ± 0.5 °C, 60% relative humidity, and a photoperiod of 14:10 h light:dark. Mortality was calculated after 24 h. All tests were performed with three biological replicates and a total of 21 leaf disks were used for the bioassay. The effect of the synergist TPP on fenpropathrin was evaluated according the method described above. The only difference was that TPP and fenpropathrin were first mixed according to the proportion of active ingredient of 3:1 (*m*/*m*). The field strain of BB was chosen to conduct the synergist-bioassay.

The sub-lethal effects of insecticides have been shown to influence population dynamics and facilitate resistance evolution by altering survival and development, fecundity, and sex ratio, etc. Thus a sub-lethal concentration of 0.818 mg/L (LC_30_, determined by bioassay) was chosen here to evaluate gene response to fenpropathrin exposure. For sub-lethal concentration exposure assay, a total of 1500 adult female mites were dipped into the solution of fenpropathrin (Sigma-Aldrich, St. Louis, MO, USA) at the concentration of LC_30_ or acetone (control). The surviving mites were collected 12 h, 24 h and 36 h, respectively, after fenpropathrin exposure for RNA extraction.

### 4.3. Total RNA Extraction and Reverse Transcription

Total RNA was extracted using RNeasy plus Micro Kit (Qiagen GmbH, Hilden, Germany) from 200 female adults (3–5 days old) of *P. citri* from susceptible and resistant strains and subsequently was treated with a gDNA elimination column supplied by the kit to remove genomic DNA. To check the quantity, the absorbance at 260 nm and the ratio of OD_260/280_ were measured with a Nanovue UV-Vis spectrophotometer (GE Healthcare, Fairfield, CT, USA). The RNA integrity was further confirmed by 1% agarose gel electrophoresis. The reverse transcription was carried out using PrimeScript 1st Strand cDNA Synthesis Kit (Takara Biotechnology Dalian Co., Ltd., Dalian, China) and the synthesized cDNA was stored at −20 °C.

### 4.4. Molecular Cloning, Identification and Phylogenetic Analysis

Besides *PcE1*, two novel CarE genes *PcE7* and *PcE9*, were selected based on the analysis of our transcriptome data. The open reading frames of the genes were amplified, respectively, using the corresponding pair of specific primers (Table S1) with the following procedure: 98 °C of initial incubation for 2 min followed by 35 cycles of 98 °C for 15 s, 60 °C for 15 s and 68 °C for 90 s; and 68 °C final extension for 10 min. The PCR products were purified from 1% agarose gel by MiniBEST Agarose Gel DNA Extraction Kit (Takara) and cloned into a pGEM-T Easy vector (Promega). Inserts were further sequenced for confirmation (BGI, Beijing, China). The sequences of CarEs from *P. citri* and *T. urticae* downloaded from the *T. urticae* genome portal website (http://bioinformatics.psb.ugent.be/orcae/overview/Tetur) were assembled by multiple sequence alignment using ClustalX [49]. Phylogenetic trees were constructed using a maximum likelihood method in MEGA 5.1, bootstrapping with 500 replicates [50].

### 4.5. RT-qPCR

RT-qPCR was subsequently carried out to determine the mRNA expression levels of the three genes from susceptible and resistant strains mites exposed to fenpropathrin. All the specific primer pairs of three *CarE* genes were designed using Primer 3.0 according to the open reading frames obtained in this study (Table S1) and *GAPDH* was used as an internal reference gene. The RT-qPCR was performed on a Stratagene Mx3000P thermal cycler (Stratagene, La Jolla, CA, USA) and a standard curve of amplification efficiency was constructed using a dilution series 1/2, 1/4, 1/8, 1/16 and 1/32. The reaction procedure was performed as follows: 95 °C for 2 min, followed by 40 cycles of 95 °C for 15 s and 60 °C for 30 s. The results were normalized to the GAPDH expression level using the 2^−ΔΔCt^ method [51]. All data were expressed as mean ± standard error (SE).

### 4.6. Functional Expression

Expression of *PcE1*, *PcE7*, *PcE9* and *GFP* in *Spodoptera frugiperda* Sf9 cells were performed using the Bac-to-Bac baculovirus expression system (Invitrogen Life Technologies, Carlsbad, CA, USA) following the manufacturer’s protocol. First, the target gene sequences were cloned into the pFastBac HTA expression vector (Invitrogen). Then, the recombinant baculovirus DNA was constructed and transfected into Sf9 cells which were cultured in suspension under serum-free conditions (SF-900 II SFM, Invitrogen) at 27 °C and 100 g. The recombinant CarEs or GFP baculovirus stock were collected and used to infect 25 mL of Sf9 cells at a density of 2 × 10^6^ cells/mL. Baculovirus-infected cells were harvested 72 h after infection by centrifugation at 2000× *g* for 10 min and resuspended in 5 mL 0.05 M PBS (pH 8.0) containing 0.1% Triton X-100, 0.5 M NaCl and 0.05% Tween 20. The homogenate was centrifuged at 10,000× *g* for 10 min and the supernatant was used as source of enzyme to evaluate the CarE-specific activity. The aforementioned recombinant baculovirus was used to generate baculovirus-infected cells for further cytotoxicity assays.

### 4.7. Enzymatic Assay

The specific activity of recombinant CarEs was measured using the spectrophotometric method as previously reported [52] with slight modifications. First, 125 μL of substrate solution, mixed with 1 μL of 0.03 M α-NA (Sinopharm Chemical Reagent, Shanghai, China), 1 μL of 10^−4^ M serine, 98 μL of 0.04 M phosphate buffer (pH 7.0), and 25 μL of enzyme source solution was mixed and incubated for 10 min at 30 °C, and then 25 μL of fast blue conjugate dye (Sinopharm Chemical Reagent) was added. The assays were conducted in 96-well microtitre plates and absorbance was determined using xMark™ Microplate Spectrophotometer (Bio-Rad, Hercules, CA, USA) at 600 nm, 30 °C for 10 min. Protein contents were measured using Bio-Rad protein assay reagent (Bio-Rad) with bovine serum albumin as a standard.

### 4.8. Cytotoxicity Assay

The cytotoxicity effect of fenpropathrin was determined using a MTT Cell Proliferation and Cytotoxicity Assay Kit (Solarbio, Shanghai, China). Cells expressing *PcE1*, *PcE7* or *PcE9* were produced by infection of Sf9 insect cells with *PcE1*-, *PcE7*-, or *PcE9*- expressed baculovirus. Parental Sf9 cells infected with *GFP*-expressed baculovirus served as control. The cytotoxic effects of fenpropathrin treatment with different concentration and time course were initially tested with Sf9 parental cells. For the assay, 500 μL of infected cells (1 × 10^5^ cells/well) was transferred into a 24-well plate and pre-incubated for 24 h. Subsequently, 10 μL of fenpropathrin diluted with acetone ranging from 1 to 100 μM (1, 5, 10, 25, 50 and 100 μM) was added to the culture and incubated for 24 h. Then, the culture medium was removed and 220 μL MTT solution (200 μL fresh culture media and 20 μL MTT) was added to each well of the plate and incubated for 4 h. Finally, MTT solution was removed and 300 μL dimethyl sulphoxide was added to each well. After mild shock for 10 min, the absorbance of formazan product was measured at 490 nm using a Mustiskan EX microtiter plate reader (BioTek, Winooski, VT, USA). Cell viability was calculated as the percentage of viable cells relative to cells treated with acetone alone. Three replications were used for each treatment.

### 4.9. RNAi Bioassay

RNAi was applied to further explore the biological functions of CarE genes in *P. citri*. *PcE1*, *PcE7* and *PcE9* were amplified by PCR using primers (Table S1) containing the T7 RNA polymerase promoter. The dsRNA were synthesized in vitro using a TranscriptAid T7 High Yield Transcription Kit (Thermo Scientific, Waltham, MA, USA) according to the manufacturer’s instructions with the purified PCR products. The dsRNA were diluted with nuclease-free water to a final concentration of 500 ng/μL. To assure the quality of synthesized dsRNA, the dsRNA products was analyzed with 1% agarose gel electrophoresis and quantified using a Nanovue UV-Vis spectrophotometer (GE Healthcare, Bucks, UK) and stored at −80 °C.

Gene silence was carried out according to the plant leaf method in our previous study [41]. The field strain of BB was chosen to conduct the RNAi bioassay. First, an 8 cm citrus tender leaflet was detached from the citrus seeding (*Aurantii fructus*) and washed with water. Then, the leaflet was incubated in oven at 60 °C for 10 min and subsequently inserted into a 250 μL Axygen nuclease-free PCR tube containing 200 μL dsRNA or nuclease-free water for 1 h recovery period. After that, 30 female adult mites from BB strain were transferred onto the leaf with a soft brush. The PCR tube with the tender leaflet was moved into a 50 mL plastic tube and covered with a piece of thin gauze tightly held with a rubber band. Finally, the devices were placed in an incubator under the condition of 25 ± 1 °C, 50% ± 5% relative humidity (RH) and a photoperiod of 14:10 h light:dark. The solution in the PCR tube was renewed daily. After incubation for two days, about 20 surviving female adults on the leaf were collected for RNA extraction. In parallel, the surviving mites after RNAi or nuclease-free water treatment were collected for insecticide bioassay. The bioassay was performed according to the procedure above. The surviving mites were transferred on the leaf-disk and then dipped into the solution of fenpropathrin (LC_50_, 4.519 mg/L) for 5 s. Twenty four hours later, the mortality was calculated to evaluate the sensitivity of mites feeding on dsRNA or nuclease-free water to fenpropathrin. The mites were determined as dead by the criteria that mites express no response to the stimulation with a soft brush. Four biological replicates were performed for each sample.

### 4.10. Statistical Analysis

All of the experiments involved at least three biological replications. The differences in expression levels among different strains and the relative quantity after fenpropathrin exposure were analyzed and the significance was determined by independent sample *t*-test with a *p* < 0.05. For the RNA interference (RNAi), the significant differences of gene expression and mortality after fenpropathrin exposure were also determined by independent sample *t*-test with a *p* < 0.05. Probit analysis was used to calculate the median lethal concentration (LC_50_) in insecticide bioassays and MTT bioassays with 95% confidence intervals. All data were analyzed using SPSS version 16.0 software (SPSS Inc., Chicago, IL, USA). In the current study, all data were given as mean ± SE.

## 5. Conclusions

In conclusion, the current study provides insights to explore the mechanism of fenpropathrin resistance through a series of biochemical and molecular approaches in *P. citri* of several field strains. The spatiotemporal expression pattern analysis found up-regulation of three *PcEs* genes after insecticide exposure and in several field resistant strains, indicating *PcEs* may play roles in tolerance to fenpropathrin. Heterologous expression combined with MTT cytotoxicity assays in Sf9 cells demonstrated that PcEs probably detoxify fenpropathrin. The reverse genetic study through leaf-mediated dsRNA feeding further support the hypothesis that PcEs may be involved in the detoxification of fenpropathrin in *P. citri*. The current data provide evidence that CarE-mediated metabolic resistance through up-regulation is more likely to be developed in modest resistant strains of *P. citri*. To further illustrate the underlying molecular mechanism of CarEs-mediated pyrethroids resistance in *P. citri*, in vitro metabolism of fenpropathrin with purified PcE1, PcE7 and PcE9 protein will be expected to conducted to clarify whether sequestration or detoxification as the major mechanism leading to fenpropathrin resistance.

## Figures and Tables

**Table 1:** Synergistic effects of TPP on fenpropathrin against *P. citri* of BB population.

Compound	LC_50_ (95% FL) (mg/L)	Slope (SE)	χ^2 a^	SR ^b^
fenpropathrin	4.339 (2.596–7.252)	0.123	1.750 *	3.09
+TPP	1.405 (0.866–2.664)	0.143	0.788 *	-

^a^ Pearson chi-square, goodness-of-fit test; ^b^ Synergism ratio = LC_50_ of fenpropathrin/LC_50_ of (TPP + fenpropathrin); * Meant pass the χ^2^ test.

## Supplementary Materials

Supplementary materials can be found at www.mdpi.com/1422-0067/17/8/1361/s1.
