# Supplementary material for: Involvement of Three Esterase Genes from Panonychus citri (McGregor) in Fenpropathrin Resistance
Source: Int J Mol Sci. 2016 Aug 19;17(8):1361. doi: 10.3390/ijms17081361 (PMC5000756; doi:10.3390/ijms17081361)
Supplement: Supplementary file 1 [file ijms-17-01361-s001.pdf]

Supplementary Material: Involvement of Three Esterase Genes from Panonychus citri (McGregor) in Fenpropathrin Resistance

Xiao-Min Shen, Chong-Yu Liao, Xue-Ping Lu, Zhe Wang, Jin-Jun Wang and Wei Dou

Table S1. Primers used for quantitative reverse transcription PCR (RT-qPCR) and double-stranded RNA (dsRNA) synthesis.

Primer Names		Sequence (5′–3′)	Product Size (bp)
ORF Cloning	PcE1-F	GGATCCGATGATATTTAAGTTGCTAATATTTGTGC	1653
	PcE1-R	CTCGAGTTAAAGCTCTTCATGGTTATCGG	
	PcE7-F	CCATGGGGATGAATTTGGAAGAGTTTAAAATGT	1692
	PcE7-R	CTGCAGTTAGTTTTCTAGTGGAAATTGGT	
	PcE9-F	GGATCCGATGAAATTAACTTTTCTTTTATTTGTG	2118
	PcE9-R	CTCGAGTTAGAAATAAACACGATACATTCTAG	
RT-qPCR	PcE1-qF	GACGATGTTCCAATGATGT	182
	PcE1-qR	ATTTCAACCTTTCCAGATTC	
	PcE7-qF	CCACTTATTTTCGCGATGGT	165
	PcE7-qR	TGATGAACGCTGATCCATGT	
	PcE9-qF	CCACCTTCAGGTCCAGTTGT	199
	PcE9-qR	ACGCTGGTTTAGGTTTGGTG	
dsRNA Synthesis	PcE1-dsF	taatacgactcactatagggAGTGTCTTCGCCTCGGTTAA	363
	PcE1-dsR	taatacgactcactatagggGTGAGTTGGACAAGCGAGTG	
	PcE7-dsF	taatacgactcactatagggTTCTCGATCCCAGTGCCATT	458
	PcE7-dsR	taatacgactcactatagggTTACCACCCGAACTTAGGCC	
	PcE9-dsF	taatacgactcactatagggCATCGAACCAACCTCACGTG	483
	PcE9-dsR	taatacgactcactatagggCGCTCCTTTAATGCCCAAGT	

ORF, open reading frame; The underlined regions are restriction enzyme sites. The lowercase letters represent the T7 promoter sequences for efficient in vitro transcription in dsRNA synthesis.
